# Whole-genome sequence of *Enterobacter hormaechei*, isolate *jjbc* recovered from the gut of *Plutella xylostella* feeding on cabbage

**DOI:** 10.1128/mra.00330-24

**Published:** 2024-07-22

**Authors:** Loretta Mugo-Kamiri, Lea Schäfer, Jörg T. Wennmann, Elisabeth A. Herniou, Ben Raymond

**Affiliations:** 1 Institut de Recherche sur la Biologie de l'Insecte, IRBI, UMR 7261, CNRS - University of Tours, Tours, France; 2 Center for Ecology and Conservation, Penryn Campus, College of Life and Environmental Science, University of Exeter, Cornwall, United Kingdom; 3 Julius Kühn Institute (JKI) – Federal Research Centre for Cultivated Plants, Institute for Biological Control, Dossenheim, Germany; California State University San Marcos, San Marcos, California, USA

**Keywords:** gut microbiota, lepidoptera, host-microbe interactions, antimicrobial resistance

## Abstract

We present the whole-genome sequence of *Enterobacter hormaechei* (previously *Enterobacter cloacae*) obtained from long and short reads. It is a dominant gut symbiont of the notorious crop pest *Plutella xylostella*, highly prevalent in lepidopteran midguts and a useful model for the evolution of resistance to antimicrobials.

## ANNOUNCEMENT

*Enterobacter* symbionts isolated from lepidopterans encode genes with functions such as digestion of plant cell wall carbohydrates, detoxification of plant allelochemicals, and the breakdown of insecticides (1 – 4), capabilities that are consistent with a niche as symbionts

of herbivores. The ease of use of *Enterobacter hormaechei* in gnotobiotic insect rearing systems and the possession of an AmpC beta-lactamase also mean that this bacterium is a valuable model for antimicrobial resistance in the *Enterobactericeae* (5).

*E. hormaechei* isolate jjbc was isolated by homogenizing *Plutella xylostella* larvae feeding on Chinese cabbage, *Brassica pekinensis*, at the Department of Zoology, University of Oxford. Homogenates were plated on Luria-Bertani (LB) agar and incubated at 30°C (6). After streaking clones, bacteria were grown in LB broth overnight at 30°C. DNA extraction from these cultures and sequencing were done at MicrobesNG (Birmingham, UK). Briefly, 40 µL of bacterial cell suspension was lysed in 120 µL of TE buffer-containing lysozyme, metapolyzyme, and RNase A. Proteinase K (final concentration 0.1 mg/mL) and SDS [final concentration 0.5% (vol/vol)] were added and incubated for 5 min at 65°C. Genomic DNA was purified using an equal volume of SPRI beads and resuspended in Elution buffer (10 mM Tris-HCl, pH 8.0). Genomic DNA libraries were prepared using the Nextera XT Library Prep Kit (Illumina, San Diego, USA) with these modifications: input DNA was increased twofold, and PCR elongation time was increased to 45 seconds. For the short reads, libraries were sequenced on a lllumina NovaSeq 6000 (Illumina, San Diego, USA) using a 250 bp paired-end protocol. For the long reads, DNA libraries were prepared using the Oxford Nanopore SQK-LSK109 and Native Barcoding EXP-NBD104/114 kits (ONT, UK) with 400–500 ng High Molecular Weight DNA, fragmented without size selection. Barcoded samples were pooled and loaded in a Library prepFLO-MIN111 (R10.3) flow cell, in a GridION (ONT, UK).

**Fig 1:**
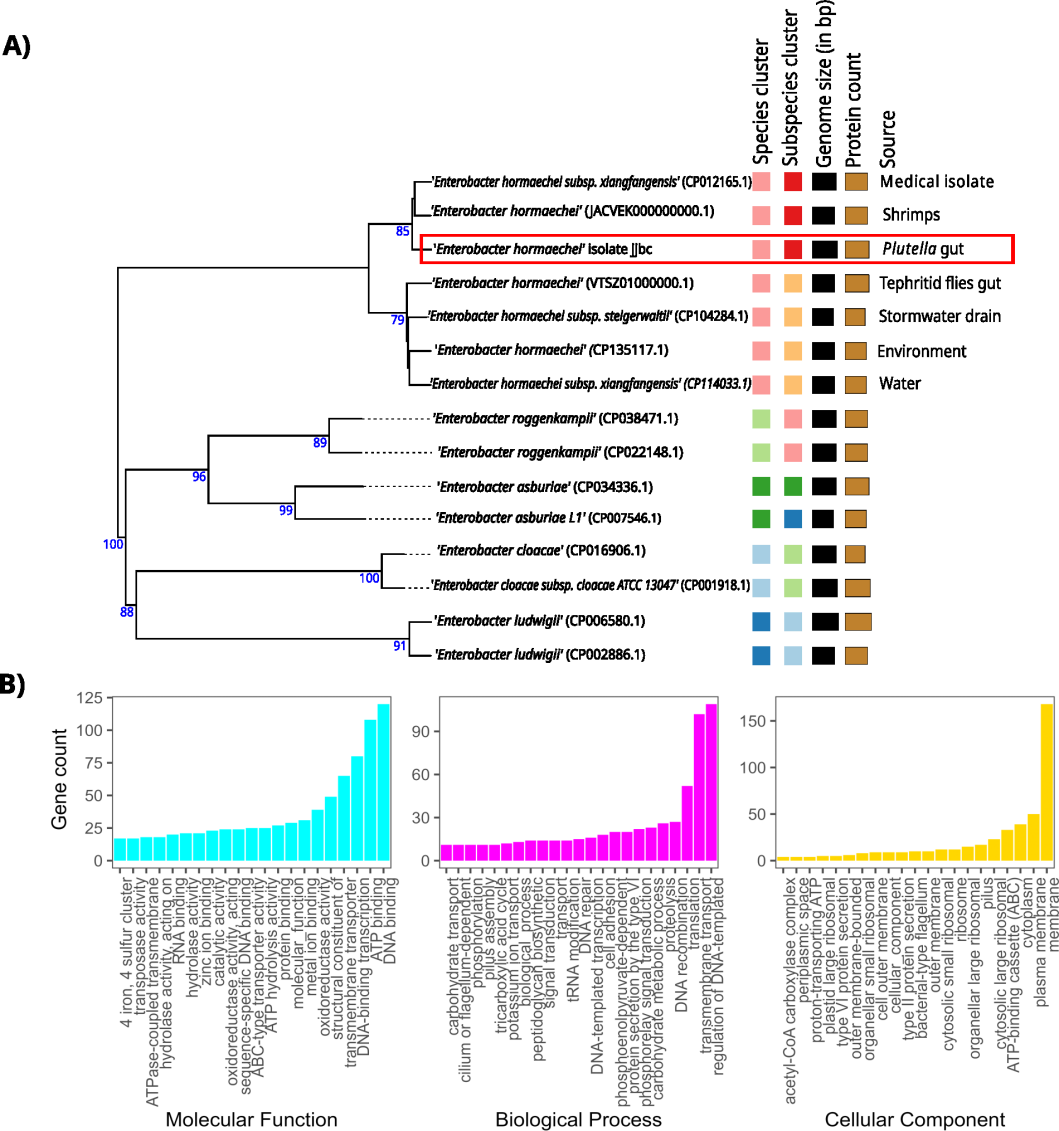
(A) Whole-genome phylogeny generated using the TYGS server confirming isolate jjbc belongs to the species *E. hormaecehei* and clusters with isolates from environmental samples, shrimps, and insect guts. Colored squares show five species clusters and seven sub-species clusters and the relative genome size and protein count of different genomes. Numbers above the branches represent branch support values inferred from 100 pseudo-bootstrap replicates based on Genome BLAST Distance Phylogeny under the algorithm distance formula (18). (B) Top 20 Gene Ontology terms showing the number of genes associated with each function for the categories of molecular function, biological process, and cellular component.

We obtained 1,172,223 Illumina reads and 13,666 ONT reads. The read quality was verified using FastQC (v. 0.12.1) (7) and NanoPlot (v. 1.42.0) (8), respectively. Genome assembly was performed using the fully automated hybrid assembly pipeline Unicycler (v. 0.5.0) (9). Default settings were used for all software (10). Manual intervention was required to resolve the following issues in the automatic assembly: (i) erroneous removal of genuine sequences during graph cleaning of the short-read assembly and (ii) collapse of inter-plasmidic repeats (11). The correction of these assembly errors resulted in a fully resolved genome consisting of one circular chromosome and four circular plasmids (Table 1), with a BUSCO completeness score of C:98.8%(S:98.6%,D:0.2%),F:0.2%,M:1.0%,*n*:440 (12) and a mean read depth of 110×. The closest matching species confirmed using the Type (Strain) Genome Server (TYGS) (13) is *E. hormaechei* (Fig. 1A). This genome was annotated using PGAP v.6.6 (14) and contains 4,951 predicted genes. Functional annotation shows multiple genes associated with carbohydrate metabolism (Fig. 1B). Using ResFinder 4.4.2 server (15), we identified two antimicrobial resistance genes: fosA for Fosfomycin resistance (96.71% alignment and 100% coverage) and blaACT-16, an AmpC beta-lactamase (99.74% alignment and 100% coverage) common in *Enterobacter* spp. (16).

**TABLE 1:** Summary of the genome assembly statistics for *E. hormaechei* isolate jjbc^*a*^

Replicon	Length (bp)	GC content (%)	Topology	GenBank accession
Chromosome	4,854,432	55.3	Circular	CP136935
Plasmid 1	87,769	50.5	Circular	CP136936
Plasmid 2	82,397	53.2	Circular	CP136937
Plasmid 3	64,969	52.4	Circular	CP136938
Plasmid 4	53,743	50.8	Circular	CP136939

^*a*^ Circularity of contigs was confirmed through Unicycler’s final assembly graph (gfa1) in Bandage (v 0.8.1) (17).

## Data Availability

This Whole-Genome Shotgun project and the annotate genome have been deposited in GenBank under the Bioproject number PRJNA1026073 and BioSample accession number SAMN39460432. The raw long and short reads are available from NCBI Sequence Read Archive (SRA) under the accession numbers SRR28014489 and SRR28014490.

## References

[1] Yang F-Y , Saqib HSA , Chen J-H , Ruan Q-Q , Vasseur L , He W-Y , You M-S . 2020. Differential profiles of gut microbiota and metabolites associated with host shift of Plutella xylostella. Int J Mol Sci 21:1–15. doi:10.3390/ijms21176283 PMC7504026 32872681

[2] Anand AAP , Vennison SJ , Sankar SG , Prabhu DIG , Vasan PT , Raghuraman T , Geoffrey CJ , Vendan SE . 2010. Isolation and characterization of bacteria from the gut of Bombyx mori that degrade cellulose, xylan, pectin and starch and their impact on digestion. J Insect Sci 10:107. doi:10.1673/031.010.10701 20874394 PMC3016902

[3] Gandotra S , Bhuyan PM , Gogoi DK , Kumar A , Subramanian S . 2018. Screening of nutritionally important gut bacteria from the lepidopteran insects through qualitative enzyme assays. Proc Natl Acad Sci India Sect B Biol Sci 88:329–337. doi:10.1007/s40011-016-0762-7

[4] Xia X , Sun B , Gurr GM , Vasseur L , Xue M , You M . 2018. Gut microbiota mediate insecticide resistance in the diamondback moth, Plutella xylostella (L.). Front Microbiol 9:25. doi:10.3389/fmicb.2018.00025 29410659 PMC5787075

[5] Manktelow CJ , Penkova E , Scott L , Matthews AC , Raymond B . 2020. Strong environment-genotype interactions determine the fitness costs of antibiotic resistance in vitro and in an insect model of infection. Antimicrob Agents Chemother 64:e01033-20. doi:10.1128/AAC.01033-20 32661001 PMC7508608

[6] Somerville J , Zhou L , Raymond B . 2019. Aseptic rearing and infection with gut bacteria improve the fitness of transgenic diamondback moth, Plutella xylostella. Insects 10:89. doi:10.3390/insects10040089 30925791 PMC6523322

[7] Andrews S . 2015. FastQC: a quality control tool for high throughput sequence data. Available from: http://www.bioinformatics.babraham.ac.uk/projects/

[8] De Coster W , D’Hert S , Schultz DT , Cruts M , Van Broeckhoven C . 2018. Sequence analysis NanoPack: visualizing and processing long-read sequencing data. Bioinformatics 34:2666–2669. doi:10.1093/bioinformatics/bty149 29547981 PMC6061794

[9] Wick RR , Judd LM , Gorrie CL , Holt KE . 2017. Unicycler: resolving bacterial genome assemblies from short and long sequencing reads. PLoS Comput Biol 13:e1005595. doi:10.1371/journal.pcbi.1005595 28594827 PMC5481147

[10] Afgan E , Baker D , Batut B , van den Beek M , Bouvier D , Cech M , Chilton J , Clements D , Coraor N , Grüning BA , Guerler A , Hillman-Jackson J , Hiltemann S , Jalili V , Rasche H , Soranzo N , Goecks J , Taylor J , Nekrutenko A , Blankenberg D . 2018. The Galaxy platform for accessible, reproducible and collaborative biomedical analyses: 2018 update. Nucleic Acids Res 46:W537–W544. doi:10.1093/nar/gky379 29790989 PMC6030816

[11] Schäfer L , Jehle JA , Kleespies RG , Wennmann JT . 2024. A practical guide and Galaxy workflow to avoid inter-plasmidic repeat collapse and false gene loss in Unicycler’s hybrid assemblies. Microb Genom 10:1–14. doi:10.1099/mgen.0.001173 PMC10868617 38197876

[12] Simão FA , Waterhouse RM , Ioannidis P , Kriventseva EV , Zdobnov EM . 2015. Genome analysis BUSCO: assessing genome assembly and annotation completeness with single-copy orthologs. Bioinformatics 31:3210–3212. doi:10.1093/bioinformatics/btv351 26059717

[13] Meier-Kolthoff JP , Göker M . 2019. TYGS is an automated high-throughput platform for state-of-the-art genome-based taxonomy. Nat Commun 10:2182. doi:10.1038/s41467-019-10210-3 31097708 PMC6522516

[14] Tatusova T , DiCuccio M , Badretdin A , Chetvernin V , Nawrocki EP , Zaslavsky L , Lomsadze A , Pruitt KD , Borodovsky M , Ostell J . 2016. NCBI prokaryotic genome annotation pipeline. Nucleic Acids Res 44:6614–6624. doi:10.1093/nar/gkw569 27342282 PMC5001611

[15] Florensa AF , Kaas RS , Clausen P , Aytan-Aktug D , Aarestrup FM . 2022. ResFinder-an open online resource for identification of antimicrobial resistance genes in next-generation sequencing data and prediction of phenotypes from genotypes. Microb Genom 8:000748. doi:10.1099/mgen.0.000748 35072601 PMC8914360

[16] Drieux L , Brossier F , Sougakoff W , Jarlier V . 2008. Phenotypic detection of extended-spectrum β-lactamase production in Enterobacteriaceae: review and bench guide. Clin Microbiol Infect 14:90–103. doi:10.1111/j.1469-0691.2007.01846.x 18154532

[17] Wick RR , Schultz MB , Zobel J , Holt KE . 2015. Bandage: interactive visualization of de novo genome assemblies. Bioinformatics 31:3350–3352. doi:10.1093/bioinformatics/btv383 26099265 PMC4595904

[18] Meier-Kolthoff JP , Auch AF , Klenk HP , Göker M . 2013. Genome sequence-based species delimitation with confidence intervals and improved distance functions. BMC Bioinformatics 14:1–14. doi:10.1186/1471-2105-14-60 23432962 PMC3665452

